# Investigating spatiotemporal and kinematic gait parameters in individuals with Parkinson’s disease with a history of freezing of gait and exploring the effects of dopaminergic therapy on freezing of gait subtypes

**DOI:** 10.3389/fnins.2024.1404613

**Published:** 2024-07-10

**Authors:** Po-Hsi Lin, Yun-Ru Lai, Chia-Yi Lien, Chih-Cheng Huang, Yi-Fang Chiang, Chien-Feng Kung, Chih-Jui Chen, Cheng-Hsien Lu

**Affiliations:** ^1^Departments of Neurology, Kaohsiung Chang Gung Memorial Hospital, Chang Gung University College of Medicine, Kaohsiung, Taiwan; ^2^Hyperbaric Oxygen Therapy Center, Kaohsiung Chang Gung Memorial Hospital, Chang Gung University College of Medicine, Kaohsiung, Taiwan; ^3^Department of Intelligent Commerce, National Kaohsiung University of Science and Technology, Kaohsiung, Taiwan; ^4^LongGood MediTech Ltd., Taipei, Taiwan; ^5^Center for Shockwave Medicine and Tissue Engineering, Kaohsiung Chang Gung Memorial Hospital, Chang Gung University College of Medicine, Kaohsiung, Taiwan; ^6^Department of Biological Science, National Sun Yat-Sen University, Kaohsiung, Taiwan; ^7^Department of Neurology, Xiamen Chang Gung Memorial Hospital, Xiamen, China

**Keywords:** freezing of gait, kinematics, levodopa-unresponsive, levodoparesponsive, Parkinson’s disease, spatiotemporal parameters

## Abstract

**Introduction:**

Freezing of Gait (FOG) is a prevalent and debilitating symptom in idiopathic Parkinson’s disease (PD). This study evaluated spatiotemporal and kinematic gait parameters in individuals with PD with a history of FOG and explored the effects of dopaminergic therapy on FOG subtypes.

**Methods:**

One hundred and nine individuals with PD underwent clinical assessments and quantitative biomechanical measures during walking cycles before and after dopaminergic therapy. Individuals with FOG were classified into levodopa-responsive and levodopa-unresponsive groups.

**Results:**

Individuals with FOG displayed longer disease duration and higher Unified Parkinson’s Disease Rating Scale (UPDRS) II, III, IV scores, and total scores and levodopa equivalent dose, than those without FOG (all *p* < 0.0001). Following propensity score matching of 15 pairs based on UPDRS total score and disease duration during the off-medication state, the analysis comparing the FOG and non-FOG groups revealed no significant differences in spatiotemporal and kinematic parameters. In 39 cases of FOG, dopaminergic therapy improved gait performance in individuals with PD, enhancing spatiotemporal parameters (speed, stride length, step length, step variability) and kinematic parameters (shoulder and elbow flexion/extension range of motion (ROM), pelvic rotation, and hip abduction/adduction ROM) regardless of FOG responsiveness to dopaminergic therapy. A significant difference in trunk sway ROM (*p* = 0.029) remained before and after dopaminergic therapy, even after adjusting for disease duration and clinical severity.

**Discussion:**

Dopaminergic therapy had varying effects on PD with FOG, improving several spatiotemporal and kinematic gait parameters but being less effective in levodopa-unresponsive cases. Quantitative biomechanical measures offer detailed insights into gait performance, aiding personalized fall risk assessment and guiding individualized rehabilitation programs.

## Introduction

Freezing of gait (FOG) manifests as abrupt, intermittent halts in movement, marked by a sensation of feet adhering to the ground and an incapacity to initiate purposeful forward steps effectively (Macht et al., 2007). This distressing symptom often culminates in falls, reduced mobility, and an overall decline in quality of life.

Dopaminergic therapy, including levodopa and dopamine agonists (DA), can improve postural instability in the early stages of Parkinson’s disease (PD); However, its effectiveness does not decrease over time; rather, disease progression due to neurodegeneration and the emergence of dopaminergic-resistant symptoms (such as gait problems and postural instability) necessitate higher doses to control symptoms (Bekkers et al., 2018).

Past evidence shows that FOG is strongly associated with increasing disease severity and longer duration of levodopa treatment (Giladi et al., 2001a). The pathophysiology of FOG involves several mechanisms, including the “levodopa paradox” (Koehler et al., 2021), the effects of different DA on various dopamine receptor subtypes (Schaafsma et al., 2003), overstimulation of dopamine receptors in frontal-subcortical circuits or frontal-striatal circuits (Cossu et al., 2015), and the mixed presence of underlying dopaminergic and nondopaminergic brain lesions (Nonnekes et al., 2016).

The use of symptom diaries and questionnaires to assess PD with FOG is limited by subjectivity, potential reliability issues due to cognitive impairments, and challenges in clinical management (Giladi et al., 2009; Nieuwboer et al., 2009). These limitations highlight the need for the development of more objective and reliable measures for assessing FOG in this patient population (Virmani et al., 2021). Despite these findings, few studies have specifically investigated the effects of dopamine on spatiotemporal parameters or kinematic variables in individuals in PD with a history of FOG (Virmani et al., 2021; Shida et al., 2023). Parameters such as step length, speed, stride variability, and joint angles are critical to understanding gait disorders in this population. Quantitative biomechanical measures of these parameters indicate walking performance throughout the gait cycle and facilitate personalized assessment of fall risk (Hubble et al., 2015).

The association between dopaminergic therapy and FOG in PD is intricate, and three main types of FOG related to levodopa therapy have been delineated: namely, levodopa-responsive, levodopa-unresponsive, and levodopa-induced FOG (Factor et al., 2014; Nonnekes et al., 2020; Nonnekes and Bloem, 2020). Levodopa-responsive FOG typically observes in the early stages of PD, where FOG improves with levodopa therapy, levodopa-unresponsive FOG occurs with disease progression, often due to involve non-dopaminergic pathways or factors such as constipation and dyskinesias that impair effective levodopa delivery and levodopa-induced FOG is characterized by the onset of FOG predominantly during the dopaminergic on-state, with minimal or no occurrence during the off-state, following levodopa administration (Factor et al., 2014; Nonnekes et al., 2020; Nonnekes and Bloem, 2020). The objective of this study is to investigate spatiotemporal and kinematic gait parameters in individuals with PD with a history of FOG and to explore the effects of dopaminergic therapy on FOG subtypes. The outcomes obtained could potentially heighten recognition of these variations among distinct phenotypes, thereby aiding in tailoring strategies for individuals afflicted with PD experiencing FOG. This understanding can subsequently inform interventions aimed at augmenting their long-term quality of life.

## Patients and methods

### Study design and patient selection

This case–control study was conducted at a tertiary medical center in southern Taiwan with 109 participants diagnosed with idiopathic PD according to clinical criteria. Inclusion criteria were participants who had a steady dose of anti-Parkinsonian agents for more than 6 months, Hoehn and Yahr stage 1–3, and could walk independently. Exclusion criteria were newly diagnosed PD, neurological signs not related to PD, advanced PD stage, mild to moderate dementia (CDR ≥1), balance interference etiologies, and follow-up of less than 6 months. The study was approved by the hospital’s Institutional Review Committees on Human Research (IRB 201901802B0), and all participants provided informed consent.

### Clinical diseases severity and subtypes of FOG in PD

A complete medical history was recorded, including age at disease onset, sex, body mass index (BMI), disease duration, and levodopa equivalent dose (LEDD) (Tomlinson et al., 2010). The “off” state was defined as 12 h after the last dose of anti-parkinsonism agents, while the “on” state was defined as at least 1 h after taking such agents (Lai et al., 2022). The clinical severity of PD was assessed using the Unified Parkinson’s Disease Rating Scale (UPDRS) and Hoehn and Yahr stages (Martinez-Martin et al., 1994). The UPDRS total score was calculated by summing the scores of subscales I, II, III, and IV. FOG was assessed using the New Freezing of Gait Questionnaire (NFOG-Q) (Nieuwboer et al., 2009). Three primary categories of FOG associated with levodopa therapy have been distinguished based on subjective patient self-reports: levodopa-responsive, levodopa-unresponsive, and levodopa-induced FOG (Nonnekes and Bloem, 2020). In this study, levodopa-responsive FOG is referred to as “off” FOG, which is alleviated by dopaminergic medication. In contrast, levodopa-unresponsive FOG is defined as FOG that occurs in both “on” and “off” states (Factor et al., 2014), or FOG that does not improve despite clinically optimized levodopa dosing (Virmani et al., 2021). Levodopa-induced FOG is characterized by the onset of FOG predominantly during the dopaminergic on-state. In our study, only levodopa-responsive and levodopa-unresponsive FOG were observed. We also calculated scores using the Cognitive Abilities Screening Instrument (CASI C-2.0), which consists of 20 items divided into 9 domains. The sum of the scores ranges from 0 to 100, with higher scores indicating better cognitive ability (Chen et al., 2017).

### Assessment of gait analysis

We utilized three-dimensional Kinect V2 detectors to automatically track skeletal data and reconstruct 25 key reference joint points at a sampling rate of 30 Hz. The system operated on a Windows 10 platform with an i5 CPU or higher and employed a specialized algorithm (GaitBEST, LongGood MediTech Ltd., Taipei, Taiwan) as described by Lai et al. (2022). This gait analysis system used Kinect detectors to automatically position the human body, capturing the locations of joint points, which were then processed by the main software program for further analysis. Key time points and displacement values were calculated from these data. Spatiotemporal and kinematic parameters during the walking gait cycle were assessed following verbal instructions to initiate gait with the most affected leg for PD patients and the right leg for healthy controls.

Position the sensor camera on a platform elevated to a height of 80 cm. Ensure that an unobstructed area measuring 2.5 meters in width and 4.5 meters in length is maintained in front of the sensor camera. The measurement walkway must be oriented perpendicular to the sensor camera. Patients were instructed to walk 4.5 meters, turn 180° after crossing a line on the ground, and return to the initial starting position (Lai et al., 2022).

To mitigate the effects of FOG during the walk cycle, we employed several methods, such as using a laser pointer to shine light in front of the patient’s foot. If FOG significantly prolonged the testing time, the system would automatically stop, and the data from that session would be excluded from the analysis. We repeated the same test in such cases. The researcher could observe the visualized variation caused by FOG on the output report, which shows the range of motion (ROM) plot during the entire walking period. We analyzed and averaged three successful trials during both the “off” and “on” phases.

### Kinematics in trunk, pelvis and four extremities during walking cycle

The shoulder joint exhibits six fundamental motions: abduction, flexion, extension, adduction, internal rotation, and external rotation. Similarly, the pelvis demonstrates six essential motions: anterior pelvic tilt, posterior pelvic tilt, left pelvic obliquity, right pelvic obliquity, left pelvic rotation, and right pelvic rotation. In the hip joint, four primary motions are observed: hip flexion, hip extension, hip abduction, and hip adduction. Conversely, the elbow and knee joints exhibit two fundamental motions: flexion and extension. Lastly, the trunk encompasses three basic ROM: bending, sway, and rotation.

### Statistical analysis

We performed propensity score matching to reduce selection bias for each spatiotemporal and kinematic parameters included in the FOG and non-FOG groups. We used a ratio of 1:1 with a caliper value set at 0.1 to ensure the comparability of individuals from both the FOG and non-FOG groups. The following variables expected to affect spatiotemporal and kinematic parameter were selected: diseases duration and UPDRS ALL (off phase). The study compared the baseline characteristics of individuals with FOG and those without FOG using an independent t-test. Given the extensive multiple comparisons conducted in this study, the Benjamini-Hochberg correction was applied to control the false discovery rate. The paired t-test was used to compare spatiotemporal and kinematic variables before and after dopaminergic therapy within each group, while the mixed model ANOVA was used to compare these variables before and after dopaminergic therapy between the two groups after controlling disease duration and UPDRS total scores as covariates. All statistical analyses were conducted using the IBM SPSS Statistics v23 software (IBM, Redmond, WA, United States).

## Results

### General characteristics of patients

In a cohort comprising 109 individuals diagnosed with PD, 39 exhibited FOG, while 70 did not. The baseline dermatological data for all participants are detailed in Table 1. Those with FOG showed higher LEDD and significantly elevated scores across various sections of the UPDRS including II, III, IV and total score compared to non-FOG individuals (all *p* < 0.0001). Furthermore, the FOG group had a higher prevalence of dyskinesia and motor fluctuations compared to the non-FOG group (both *p* < 0.0001).

**Table 1:** Baseline characteristics of Parkinson’s disease.

	Before propensity score matching		*P*-value	After propensity score matching		*P*-value
	FOG (*n* = 39)	Non-FOG (*n* = 70)		FOG (*n* = 15)	Non-FOG (*n* = 15)	
Age, years	66.1 ± 9.0	69.6 ± 9.7	0.06	64.2 ± 8.2	73.13 ± 7.5	0.004*
Sex (male/female)	18/21	36/34	0.84	8/7	7/8	0.72
Height (m)	1.6 ± 0.1	1.6 ± 0.1	0.74	1.6 ± 0.1	1.6 ± 0.1	0.23
Body weight (kg)	61.5 ± 11.7	64.3 ± 11.3	0.21	61.0 ± 9.8	62.1 ± 9.6	0.76
Body mass index (kg/m^2^)	24.4 ± 4.4	25.3 ± 3.9	0.25	23.6 ± 3.6	25.2 ± 4.9	0.31
Waist circumference (cm)	86.9 ± 9.7	90.3 ± 9.9	0.08	86.9 ± 9.0	90.5 ± 9.4	0.29
Disease duration, years	7.9 ± 4.7	3.4 ± 2.4	<0.0001*	7.6 ± 5.8	6.7 ± 3.9	0.63
UPDRS I (off phase)	1.9 ± 1.3	1.6 ± 1.4	0.21	1.9 ± 1.1	2.7 ± 1.9	0.22
UPDRS II (off phase)	14.8 ± 3.9	6.9 ± 3.9	<0.0001*	13.3 ± 4.0	10.8 ± 5.0	0.15
UPDRS III (off phase)	23.9 ± 10.7	14.7 ± 7.3	<0.0001*	18.4 ± 7.1	21.0 ± 7.1	0.32
UPDRS IV	3.3 ± 3.1	0.7 ± 0.4	<0.0001*	3.4 ± 2.7	2.4 ± 1.7	0.41
UPDRS ALL (off phase)	43.8 ± 13.3	23.9 ± 11.4	<0.0001*	36.9 ± 9.9	36.9 ± 11.6	1.0
LEDD (mg)	1258.2 ± 573.7	531.1 ± 357.4	<0.0001*	1216.2 ± 646.6	780.0 ± 395.5	0.04
Hoehn and Yahr stages	2.6 ± 0.9	1.8 ± 0.8	<0.0001*	2.6 ± 0.9	2.1 ± 0.8	0.17
Dyskinesia	17	3	<0.0001*	8	3	0.06
Motor fluctuation	23	5	<0.0001*	10	4	0.03*
“Off” dystonia	13	13	0.06	6	6	1.0
Cognitive Abilities Screening Instrument	85.3 ± 13.5	81.6 ± 13.2	0.22	82.7 ± 18.6	71.2 ± 15.6	0.11

*Indicates that *p*-value < 0.05; Data are presented as mean+/-SD or number. UPDRS, Unified Parkinson’s Disease Rating Scale; LEDD, Levodopa equivalent dose.

### Baseline spatiotemporal and kinematic variables

Table 2 presents the spatiotemporal and kinematic parameters observed during the walking gait cycle in individuals diagnosed with PD with and without FOG. Significant differences were observed in spatiotemporal and kinematic parameters between PD with and without FOG. The spatiotemporal parameters showing significant differences included speed (m/s), stride length (m), step length (m), step length variability (CV), step time variability (CV), and turning speed (m/s) (*p* = 0.003, *p* < 0.0001, *p* < 0.0001, *p* = 0.02, *p* = 0.03, and *p* = 0.009, respectively). Additionally, significant differences in the kinematic parameters of shoulder Flexion (Flex)/extension (Ext) and abduction (Abd) and adduction (Add) ROM, hip flexion/extension ROM (°) and knee flexion/extension ROM (°) (*p* < 0.0001, *p* = 0.01, *p* = 0.02 and *p* = 0.02, respectively) were identified by Benjamini-Hochberg correction. Following propensity score matching of 15 pairs based on UPDRS total score during the off-medication state and disease duration, the analysis revealed no significant differences in spatiotemporal and kinematic parameters between the FOG and non-FOG groups during the off-medication state.

**Table 2:** Baseline spatiotemporal and kinematic variables between PD and healthy control.

	Before propensity score matching		Adjusted *p*-value^†^	After propensity score matching		Adjusted *p*-value^†^
	FOG (*n* = 39)	Non-FOG (*n* = 70)		FOG (*n* = 15)	Non-FOG (*n* = 15)	
Spatiotemporal parameters (off phase)						
Straight forward						
Cadence(steps/s)	1.84 ± 0.22	1.82 ± 0.16	0.71	1.84 ± 0.15	1.77 ± 0.12	0.82
Speed(m/s)	0.77 ± 0.32	0.96 ± 0.25	0.003	0.80 ± 0.26	0.80 ± 0.19	0.94
Stride length(m)	0.83 ± 0.29	1.05 ± 0.23	<0.0001*	0.86 ± 0.26	0.9 ± 0.17	0.87
Step length(m)	0.41 ± 0.15	0.53 ± 0.11	<0.0001*	0.43 ± 0.13	0.45 ± 0.09	0.87
Step length variability (CV)	20.30 ± 11.07	15.80 ± 6.67	0.02*	18.7 ± 8.72	16.77 ± 6.59	0.87
Stride Time (sec)	1.11 ± 0.17	1.099 ± 0.10	0.71	1.10 ± 0.10	1.11 ± 0.11	0.94
Step Time (sec)	0.56 ± 0.09	0.56 ± 0.05	0.82	0.55 ± 0.05	0.56 ± 0.04	0.82
Step Time variability (CV)	18.01 ± 12.26	12.74 ± 5.69	0.03*	18.26 ± 12.01	12.94 ± 4.84	0.82
Turning						
Turning time (sec)	3.89 ± 3.92	2.56 ± 1.04	0.11	3.36 ± 1.27	2.96 ± 1.11	0.41
Turning speed (m/s)	0.55 ± 0.22	0.71 ± 0.26	0.009*	0.54 ± 0.17	0.69 ± 0.3	0.41
Turning step length (m)	0.40 ± 0.08	0.43 ± 0.09	0.18	0.39 ± 0.08	0.41 ± 0.1	0.41
Kinematics (off phase)						
Shoulder Flex/Ext ROM (°)	11.87 ± 7.75	16.0 ± 11.03	<0.0001*	11.68 ± 4.87	17.12 ± 5.36	0.13
Shoulder Abd/Add ROM (°)	3.87 ± 2.32	5.43 ± 3.42	0.01*	3.57 ± 1.1	5.65 ± 3.54	0.15
Elbow Flex/Ext ROM (°)	9.28 ± 4.30	10.63 ± 6.58	0.05	9.92 ± 5.69	11.35 ± 6.41	0.89
Trunk bending ROM (°)	3.31 ± 1.22	3.54 ± 1.07	0.33	3.18 ± 0.92	3.26 ± 1.06	0.89
Trunk sway ROM (°)	3.47 ± 1.08	3.54 ± 1.38	0.79	3.30 ± 0.92	3.85 ± 1.88	0.88
Trunk rotation ROM (°)	7.97 ± 2.52	9.97 ± 3.66	0.05	8.93 ± 1.93	9.44 ± 2.41	0.89
Pelvic tilt ROM (°)	3.29 ± 1.26	3.52 ± 1.19	0.37	3.16 ± 0.87	3.24 ± 1.31	0.89
Pelvic obliquity ROM (°)	6.63 ± 1.92	6.41 ± 1.95	0.79	6.52 ± 1.7	6.41 ± 2.08	0.89
Pelvic rotation ROM (°)	7.38 ± 2.83	8.94 ± 3.79	0.20	8.88 ± 1.3	7.35 ± 2.45	0.15
Hip Flex/Ext ROM (°)	36.24 ± 10.68	42.9 ± 10.3	0.02	35.75 ± 11.44	36.45 ± 7.5	0.89
Hip Abd/Add ROM (°)	7.53 ± 2.32	8.79 ± 2.52	0.05	7.01 ± 1.48	8.86 ± 2.59	0.15
Knee Flex/Ext ROM (°)	47.61 ± 7.08	50.19 ± 7.38	0.02*	47.2 ± 9.74	48.82 ± 7.9	0.89

*Indicates that *p*-value < 0.05, † = Adjusted *p*-value is calculated by Benjamini-Hochberg correction. CV, coefficient of variation; ROM (degree), range of motion; degree = °.

### Impact of dopaminergic therapy on spatiotemporal and kinematic variances across FOG subtypes

**Figure 1:**
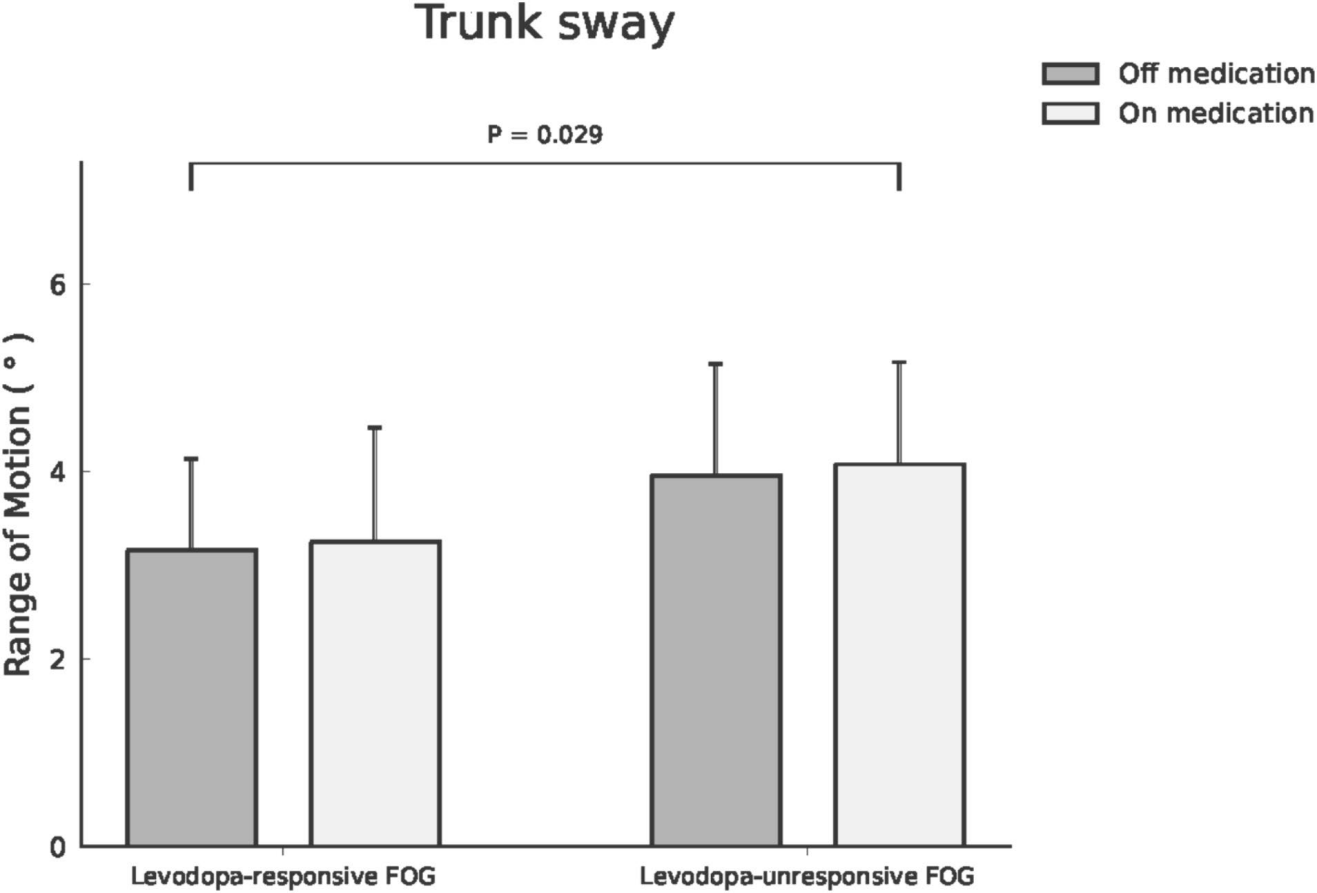
Trunk sway ROM was compared between patients with levodopa-responsive FOG and those with levodopa-unresponsive FOG, both before and after dopaminergic therapy.

Within the cohort of 39 participants affected by FOG, 24 cases were classified as levodopa-responsive FOG, while the remaining 15 cases were classified as levodopa-unresponsive FOG. Regarding the spatiotemporal and kinematic parameters distinguishing levodopa-responsive from levodopa-unresponsive FOG during the off-medication phase, gait parameters tended to be worse in the levodopa-unresponsive group, though these differences were not statistically significant. An investigation into the influence of dopaminergic therapy on spatiotemporal and kinematic variables between these distinct groups was conducted, with detailed results provided in Table 3. In the context of levodopa-responsive FOG group, parameters such as straight speed (m/s), stride length (m), step length (m), turning speed (m/s), and turning step length (m) revealed significant increases (*p* < 0.0001, *p* < 0.0001, *p* < 0.0001, *p* = 0.043, and *p* = 0.017, respectively). Moreover, step length variability decreased significantly within the levodopa-responsive FOG group. Additionally, significant enhancements were observed in shoulder flexion/extension ROM (°), shoulder abduction/adduction ROM (°), elbow flexion/extension ROM (°), hip flexion/extension ROM (°) and Abd/Add ROM (°), knee flexion/extension ROM (°), and trunk rotation ROM (°) after dopaminergic therapy (*p* < 0.0001, *p* < 0.0001, *p* < 0.0001, *p* = 0.002, *p* < 0.0001, *p* < 0.0001, and *p* < 0.0001, respectively). Conversely, in the context of levodopa-unresponsive FOG group, parameters such as cadence (steps/s), straight speed (m/s), stride length (m), and step length (m) displayed significant increases (*p* = 0.044, *p* = 0.023, *p* = 0.016, *p* = 0.016, and *p* = 0.017, respectively) following treatment. Similarly, a decrease in step length variability (*p* = 0.007) was noted within this group. Furthermore, significant improvements were evidenced in shoulder flexion/extension ROM (°), elbow flexion/extension ROM (°), hip abduction/adduction ROM (°), knee flexion/extension ROM (°), and pelvic rotation ROM (°) after dopaminergic therapy (*p* = 0.027, *p* = 0.024, *p* = 0.043, and *p* = 0.011, respectively). Significant differences in trunk sway (*p* = 0.029) were observed between the groups with levodopa-responsive FOG and those with levodopa-unresponsive FOG, both before and after dopaminergic therapy, after adjusting for disease duration and total UPDRS scores as covariates (Figure 1). The *p*-values for the significance of the between-groups factor, within-group factor, and the interaction between these two factors for each spatiotemporal and kinematic parameter in the mixed model ANOVA are presented in Table 3.

**Table 3:** Effect of dopamine replacement therapy on spatiotemporal and kinematic variables during the walking gait cycle.

	levodopa-responsive FOG (*N* = 24)		levodopa-unresponsive FOG (*N* = 15)		*P*-value for mixed model ANOVA		
	Off medication	On medication	Off medication	On medication	*P*-value^α^	*P*-value^β^	*P*-value ^γ^
Age, years	65.63 ± 8.56		66.80 ± 9.80				
Disease duration, years	8.7 ± 5.2		6.4 ± 3.3				
LEDD (mg)	1290.9 ± 555.6		1202.2 ± 620.5				
UPDRS III (off phase)	22.3 ± 11.5		26.6 ± 8.9				
UPDRS ALL (off phase)	43.5 ± 13.7		44.5 ± 14.9				
Spatiotemporal parameters							
Straight forward							
Cadence(steps/s)	1.87 ± 0.17	1.92 ± 0.20	1.77 ± 0.27	1.89 ± 0.19*	0.855	0.66	0.613
speed (m/s)	0.81 ± 0.28	1.04 ± 0.22**	0.71 ± 0.36	0.87 ± 0.21*	0.727	0.013	0.865
Stride length(m)	0.85 ± 0.27	1.08 ± 0.20**	0.78 ± 0.33	0.93 ± 0.22*	0.827	0.01	0.894
Step length(m)	0.43 ± 0.14	0.54 ± 0.10**	0.39 ± 0.16	0.46 ± 0.11*	0.818	0.008	0.898
Step length variability (CV)	17.02 ± 7.48	13.34 ± 4.04*	25.45 ± 13.87	16.67 ± 6.75**	0.213	0.451	0.134
Stride time(s)	1.08 ± 0.11	1.05 ± 0.11	1.17 ± 0.23	1.08 ± 0.12*	0.598	0.59	0.519
Step time(s)	0.54 ± 0.05	0.53 ± 0.06	0.59 ± 0.12	0.54 ± 0.06*	0.7	0.768	0.628
Step time variability (CV)	15.62 ± 10.05	11.60 ± 3.78	21.76 ± 14.72	15.60 ± 6.84	0.385	0.79	0.993
Swing phase	0.39 ± 0.04	0.40 ± 0.04	0.44 ± 0.09	0.40 ± 0.05	0.93	0.55	0.409
Stance phase	0.69 ± 0.08	0.66 ± 0.07	0.74 ± 0.15	0.68 ± 0.09	0.49	0.485	0.603
Double support time	0.29 ± 0.08	0.26 ± 0.04	0.33 ± 0.10	0.29 ± 0.05	0.605	0.736	0.413
Single support time	0.78 ± 0.08	0.79 ± 0.07	0.87 ± 0.17	0.79 ± 0.11	0.842	0.811	0.431
Pre-swing phase	0.15 ± 0.04	0.13 ± 0.02	0.17 ± 0.05	0.14 ± 0.03	0.555	0.597	0.358
Loading response time (sec)	0.15 ± 0.04	0.14 ± 0.03	0.17 ± 0.05	0.14 ± 0.03	0.467	0.411	0.418
Turning							
Turning time (sec)	4.07 ± 4.80	2.04 ± 0.44	3.59 ± 1.83	3.27 ± 2.08	0.17	0.135	0.69
Turning speed (m/s)	0.56 ± 0.25	0.68 ± 0.19*	0.55 ± 0.18	0.63 ± 0.37	0.747	0.605	0.913
Turning step length (m)	0.40 ± 0.08	0.46 ± 0.12*	0.41 ± 0.06	0.46 ± 0.14	0.586	0.879	0.99
Kinematics parameters							
Shoulder Flex/Ext ROM (°)	10.40 ± 8.41	22.98 ± 15.45**	15.09 ± 5.03	20.27 ± 9.0*	0.498	0.169	0.789
Shoulder Abd/Add ROM (°)	3.63 ± 2.69	6.50 ± 4.30**	4.39 ± 1.07	5.51 ± 2.09	0.978	0.428	0.304
Elbow Flex/Ext ROM (°)	8.60 ± 4.30	16.48 ± 10.82**	10.79 ± 4.13	12.36 ± 4.36*	0.271	0.081	0.33
Trunk bending ROM (°)	3.49 ± 1.27	3.47 ± 1.10	2.90 ± 1.03	2.97 ± 1.03	0.167	0.856	0.741
Trunk sway ROM (°)	3.16 ± 0.98	3.25 ± 1.22	3.96 ± 1.19	4.08 ± 1.09	0.289	0.904	**0.029**ǂ
Trunk rotation ROM (°)	8.08 ± 2.33	10.77 ± 3.32**	7.72 ± 3.02	9.13 ± 3.50ǂ	0.098	0.287	0.306
Pelvic tilt ROM (°)	3.45 ± 1.22	3.43 ± 0.98	2.95 ± 1.36	2.97 ± 0.96	0.178	0.559	0.905
Pelvic obliquity ROM (°)	6.31 ± 2.04	6.75 ± 1.86	7.35 ± 1.49	7.19 ± 1.73	0.279	0.536	0.224
Pelvic rotation ROM (°)	7.75 ± 2.50	9.03 ± 3.60*	6.58 ± 3.48	8.29 ± 3.40*	0.087	0.813	0.799
Hip Flex/Ext ROM (°)	34.10 ± 10.53	43.47 ± 8.17**	40.94 ± 9.89	40.61 ± 7.59	0.258	0.001	0.365
Hip Abd/Add ROM (°)	7.47 ± 2.30	8.50 ± 2.45**	7.69 ± 2.49	8.06 ± 2.58*	0.298	0.129	0.584
Knee Flex/Ext ROM (°)	44.54 ± 7.08	50.24 ± 6.63**	48.83 ± 10.60	49.27 ± 4.39	0.195	0.084	0.784

Paired-*t* test: *Indicates that *p*-value < 0.005; ** indicates that p-value < 0.001; *α* = *P*-value for within-group factor; β = *P*-value for interaction between the two factors; γ = *P*-value for between-groups factors. Mixed model ANOVA: ǂ Indicates that *p*-value < 0.05; Abbreviations: UPDRS=Unified Parkinson’s Disease Rating Scale; LEDD = Levodopa equivalent dose; CV = coefficient of variation; ROM (degree) = range of motion; degree = °.

## Discussion

### Major findings of our study

The study provides empirical evidence and presents three major findings: First, individuals with PD who exhibit FOG require higher medication dosages, experience greater clinical severity, and have a higher incidence of motor complications, such as dyskinesia and motor fluctuations, compared to those without FOG. Following propensity score matching of 15 pairs based on UPDRS total score during the off-medication state and disease duration, the analysis revealed no significant differences in spatiotemporal and kinematic parameters between the FOG and non-FOG groups during the off-medication state. Second, dopaminergic therapy improves gait performance in PD with FOG. It enhances spatiotemporal (speed, stride length, step length, step length variability) and kinematic (shoulder and elbow Flex/Ext ROM, and pelvic rotation and hip Abd/Add ROM) parameters, regardless of FOG responsiveness to levodopa. However, it is less effective in levodopa-unresponsive cases. Third, although baseline demographic data were similar between individuals with levodopa-responsive and unresponsive FOG, gait parameters tended to be worse in the levodopa-unresponsive group during the off-medication state, albeit without statistical significance. However, a substantial disparity in trunk sway persisted before and after dopaminergic therapy, even after adjusting for disease duration and clinical severity.

### Spatiotemporal and kinematic parameters in PD: fog vs. non-FOG history

One study showed that PD with FOG had increased variability in foot strike suggesting that in addition to stride length variability (Shah et al., 2018). Another study showed that while the average stride time was similar in individuals with and without FOG, stride-to-stride variability was significantly higher in PD with FOG compared to those without FOG (Hausdorff et al., 2003). Based on the literature and the characteristics of FOG in PD, the three most important spatiotemporal gait parameters for evaluating FOG are stride length, speed, and gait (step length and time) variability (Hausdorff et al., 2003; Chee et al., 2009; Shah et al., 2018). Our findings showed that, after propensity score matching, step length variability and step time variability exhibited a higher trend but did not reach statistical significance in PD patients with FOG compared to those without FOG. Regarding kinematic parameters, one study evaluated the effects of FOG on the kinematic parameters of lower limb gait in individuals with PD. The study found that FOG predominantly affects distal joints, such as the ankle and knee. PD with FOG exhibited specific kinematic differences, including greater knee flexion at initial contact, and altered ankle motion during various gait cycle phases (Shida et al., 2023). The distinct control mechanisms of the cortico-subcortical system, encompassing the basal ganglia and cortex for distal limb movements, and the reticulospinal system primarily governing pelvic motion, may contribute to an elevated anterior pelvic tilt. This intricate interplay among different neural systems underscores the complexity of coordinating movement patterns, particularly in the context of FOG in PD (Shida et al., 2023). Except for the poor reliability in ankle detection (Eltoukhy et al., 2017), clinical studies have demonstrated good validity of the kinematic measurements using the Kinect-based systems we employed (Latorre et al., 2019). Our study did not assess ankle ROM. However, our findings indicate that, after propensity score matching, hip flexion/extension and abduction/adduction ROM exhibited a lower trend but did not reach statistical significance in PD patients with FOG compared to those without FOG.

In typical walking, the pelvis moves forward on the active leg’s side, causing rotational motion in the thoracic area, coupled with either trunk counter-rotation or the opposite arm’s forward swing (Lamoth et al., 2002). As walking speed rises, the alternating rotations between the thorax and pelvis gradually synchronize in an antiphase manner. The axial rotation of the head and trunk during walking plays a fundamental role in changing one’s direction. Yet, individuals affected by PD face difficulties while turning, often displaying a tendency to move as a cohesive unit when shifting between turning and walking straight (Huxham et al., 2008). This phenomenon was particularly noticeable among our PD patients experiencing FOG compared to those without FOG, although it did not reach statistical significance after propensity score matching.

During typical walking, arm swing is a coordinated movement controlled by the central nervous system, linked with lower limb and upper limb ROM, trunk motion, and gait speed (Mirelman et al., 2015; Navarro-Lopez et al., 2022). Reduced and uneven arm swing serves as a hallmark of PD. Among our PD patients, we observed diminished arm swing and reduced range of motion (ROM) in both upper extremities (shoulder and elbow) and lower extremities (hip and knee). However, these differences did not reach statistical significance after propensity score matching.

### Dopaminergic therapy effects on FOG subtype gait cycles

Previous studies have indicated that levodopa can enhance the slower and reduced spatial aspects of gait by increasing step length and ambulation speed (Smulders et al., 2016). Additionally, levodopa appears to reduce gait variability (Hausdorff et al., 2003) and improve the kinematic and kinetic parameters of lower extremity gait in individuals with PD experiencing FOG (Shida et al., 2023).

Trunk rotation plays a crucial role in PD with FOG. During turning movements, the position of the center of mass is managed by balancing the trunk and the swinging leg on the supporting hip. Individuals with PD experiencing FOG frequently experience difficulties in trunk rotation and coordination, resulting in poor intersegmental coordination during turning. This deficiency in trunk rotation and coordination can contribute to freezing episodes, particularly when trunk movement is necessary to perform the motor task (Palmisano et al., 2024).

Our research further demonstrated significant improvements in speed (m/s), stride and step length (m), step length variability, and the ROM in shoulder, elbow, hip joints, and pelvic rotation after dopamine replacement therapy among individuals exhibiting levodopa-responsive and levodopa-unresponsive FOG phenotypes. This therapeutic intervention appears to augment speed and stride length while reducing step length variability, potentially associated with increased hip ROM, resulting in a more efficient gait pattern. It is worth considering that theories in research propose that diminished variability in step may not consistently signify a favorable outcome, as a more diverse system might offer a better response to unexpected disturbances (Curtze et al., 2015).

Turning during the walking cycle poses challenges, particularly requiring a sequence of gait initiations (Kung et al., 2023). Individuals with PD, especially those experiencing FOG, often encounter difficulties when initiating turns (Gao et al., 2020; Kung et al., 2023). Our study revealed that both the levodopa-responsive and levodopa-unresponsive FOG phenotypes exhibited improvements in turning speed and turning step length.

### Risk factors, dopaminergic drug effects, and pathophysiology of FOG

Previously identified risk factors for FOG in PD include longer disease duration, greater motor disability, higher non-tremor scores or postural instability gait disturbance phenotype, increased levodopa dosage, motor fluctuations, psychiatric features such as hallucinations, and cognitive dysfunction, particularly executive impairment (Giladi et al., 2001a; Lichter et al., 2021; Wang et al., 2022). Our study found that patients with FOG also had longer disease duration and higher UPDRS II, III, IV, and total scores, as well as higher levodopa equivalent doses compared to those without FOG. Additionally, FOG patients exhibited a higher prevalence of motor fluctuations and dyskinesia.

Researchers reviewing medical textbooks and papers from before 1972 found that FOG was significantly scarce before the introduction of levodopa, and episodes increased after its long-term use. The “levodopa paradox, “discussed by Koehler et al. (2021), highlights the complex relationship between dopaminergic therapy and FOG. While levodopa and other dopaminergic medications alleviate many motor symptoms of PD, their impact on FOG is inconsistent. Some patients experience significant improvement (Schaafsma et al., 2003), while others remain refractory to treatment or even exhibit worsened FOG (Barbeau, 1971; Ambani and Van Woert, 1973).

In addition to the “levodopa paradox” discussed by Koehler et al. (2021). Giladi et al. (2001a) indicated that FOG episodes could be exacerbated during “off “periods and not fully alleviated during “on” periods, even with optimal dopaminergic therapy. Differences in the effects of dopaminergic treatment on FOG may suggest that the D1 receptor subtype plays an important role in the efficacy of levodopa on FOG (Schaafsma et al., 2003). Recent studies indicate that some DAs may increase FOG frequency in both early (Ahlskog et al., 1992) and advanced stages of PD (Giladi et al., 2001b), whereas apomorphine appears to improve FOG (Corboy et al., 1995). This discrepancy may be attributed to dopamine receptor subtype specificity, with DAs such as ropinirole, pramipexole, and pergolide primarily targeting D2 receptors, while apomorphine targets both D1 and D2 receptors (Arnt et al., 1988). FOG in PD involves complex interactions between dopaminergic and non-dopaminergic pathways, resulting in unpredictable manifestations under different medication states (Nonnekes et al., 2016).

The theory proposes that overstimulation of dopamine receptors in frontal-subcortical circuits by peak-dose oral levodopa therapy may lead to abnormal motor responses, including FOG. Additionally, overstimulation of frontal-striatal circuits may disrupt normal gait patterns and exacerbate FOG symptoms (Cossu et al., 2015). The pathophysiology includes dysfunction in neural circuits such as the basal ganglia and prefrontal cortex, modulated by sensory feedback, contributing to the occurrence of FOG symptoms (Cossu et al., 2015). Although our patients with FOG had longer disease duration, greater clinical severity, and higher levodopa equivalent doses, dopaminergic therapy had varying effects on spatiotemporal and kinematic gait parameters. The relationship between the presence of FOG and the effects of medication remains unclear.

### Rehabilitation programs targeting freezing of gait

One study focused on levodopa-unresponsive group and demonstrated that, although levodopa is less effective in this group, dose titration using objectively measured spatiotemporal gait parameters offers a comprehensive and objective assessment. This approach provides additional insights into the biomechanical complexities of gait dysfunction (Virmani et al., 2021). The other study on predicting the responsiveness of gait variables to rehabilitation training in PD demonstrated that individuals with both early and advanced stages PD exhibit gait impairments, including reduced gait speed, step length, lower limb joint ROM, trunk rotation, and increased cadence. After a 10-week rehabilitation program, all impaired gait parameters improved, with some, like the spatial asymmetry index and trunk rotation ROM, fully normalizing (Serrao et al., 2019). This study highlights the importance of rehabilitation, particularly for the levodopa-unresponsive group. Furthermore, individuals with PD can be referred to physiotherapy for interventions addressing FOG. These interventions, such as training in cueing strategies, are recommended in the European Physiotherapy Guideline for Parkinson’s Disease (Domingos et al., 2018).

### Study limitations

Our study is subject to several limitations. Firstly, the identification and classification of FOG subtypes relied on subjective patient self-reports rather than objective assessments, potentially introducing an element of inconsistency in the classification process. Secondly, although we employed diverse methods to mitigate the impact of FOG during the walk cycle and excluded data points that significantly prolonged testing times, our research predominantly reflects the baseline neurological condition. Thirdly, the exclusion of individuals with advanced PD, who were unable to walk independently due to safety concerns and limitations associated with gait analysis tools, may have resulted in the omission of insights pertaining to the subgroup at the highest risk of falls-a critical consideration in PD research.

## Conclusion

Our findings indicate that dopaminergic therapy had varying effects on PD with FOG, improving several spatiotemporal and kinematic gait parameters but being less effective in levodopa-unresponsive group. Quantitative biomechanical measures of these parameters provide detailed insights into walking performance throughout the gait cycle, facilitating personalized assessment of fall risk. These measures can also guide the development of individualized rehabilitation programs, particularly for the levodopa-unresponsive group.

## Data availability statement

The raw data supporting the conclusions of this article can be obtained from the corresponding author upon reasonable request.

## Ethics statement

The studies involving humans were approved by the Institutional Review Board of Chang Gung Medical Foundation. The studies were conducted in accordance with the local legislation and institutional requirements. Written informed consent for participation in this study was provided by the participants’ legal guardians/next of kin.

## Author contributions

P-HL: Writing – original draft. Y-RL: Writing – original draft. C-YL: Data curation, Investigation, Writing – review & editing. C-CH: Data curation, Investigation, Writing – review & editing. Y-FC: Data curation, Investigation, Writing – review & editing. C-FK: Data curation, Investigation, Writing – review & editing. C-JC: Software, Writing – review & editing. C-HL: Conceptualization, Data curation, Formal analysis, Funding acquisition, Investigation, Methodology, Project administration, Resources, Software, Supervision, Validation, Visualization, Writing – review & editing.
